# A Multivariate Model of Stakeholder Preference for Lethal Cat Management

**DOI:** 10.1371/journal.pone.0093118

**Published:** 2014-04-15

**Authors:** Dara M. Wald, Susan K. Jacobson

**Affiliations:** 1 Center for Policy Informatics, School of Public Affairs, Arizona State University, Phoenix, Arizona, United States of America; 2 Department of Wildlife Ecology and Conservation, University of Florida, Gainesville, Florida, United States of America; University of Illinois at Urbana-Champaign, United States of America

## Abstract

Identifying stakeholder beliefs and attitudes is critical for resolving management conflicts. Debate over outdoor cat management is often described as a conflict between two groups, environmental advocates and animal welfare advocates, but little is known about the variables predicting differences among these critical stakeholder groups. We administered a mail survey to randomly selected stakeholders representing both of these groups (n = 1,596) in Florida, where contention over the management of outdoor cats has been widespread. We used a structural equation model to evaluate stakeholder intention to support non-lethal management. The cognitive hierarchy model predicted that values influenced beliefs, which predicted general and specific attitudes, which in turn, influenced behavioral intentions. We posited that specific attitudes would mediate the effect of general attitudes, beliefs, and values on management support. Model fit statistics suggested that the final model fit the data well (CFI = 0.94, RMSEA = 0.062). The final model explained 74% of the variance in management support, and positive attitudes toward lethal management (humaneness) had the largest direct effect on management support. Specific attitudes toward lethal management and general attitudes toward outdoor cats mediated the relationship between positive (p<0.05) and negative cat-related impact beliefs (p<0.05) and support for management. These results supported the specificity hypothesis and the use of the cognitive hierarchy to assess stakeholder intention to support non-lethal cat management. Our findings suggest that stakeholders can simultaneously perceive both positive and negative beliefs about outdoor cats, which influence attitudes toward and support for non-lethal management.

## Introduction

Public acceptance of wildlife management is dependent upon complex interactions between individual values, attitudes, beliefs and social interactions[1]. An understanding of the social and cognitive determinants of management support, whether efforts are aimed at managing native, domestic, or invasive species, is necessary to reduce conflict over management methods and identify management approaches with broad stakeholder support [2]–[4].

Stakeholders are individuals/groups with an interest or stake in an issue or management concern [5]. Stakeholders fundamentally influence the success of management initiatives and the implementation of public policies [6]. Stakeholders disagree over whether current approaches to managing outdoor cats (*Felis catus*) are appropriate, effective or humane [7]. Traditional management approaches for outdoor cats include lethal control, such as trapping followed by euthanasia in an animal shelter. Some animal welfare advocates consider feral cats to be “healthy wildlife” [8] and advocate non-lethal methods of control [7], [9]–[11]. Many animal welfare advocates, opposed to the use of lethal cat control, have strongly advocated for the use of non-lethal management methods, primarily Trap-Neuter-Return (TNR), which requires capture and surgical sterilization of the animal followed by return to the cats’ previous neighborhood or home range. Debate about the use of lethal or non-lethal control has led to a sharp division between stakeholder groups that has incited rancorous debate and litigation [12]. On the other side of this divide are environmental advocates, who view cats as “exotic” animals, and for whom the risks associated with outdoor/feral cats are unacceptable [2], [13]. Both groups include stakeholders with diverse perspectives about how to manage, confine, and/or control outdoor cats [14].

Researchers who have studied conflict over outdoor cats have typically evaluated the acceptability of specific management methods (e.g., removal, euthanasia, TNR) [15]–[17]. Additional studies have explored the role of socio-demographic and cognitive variables on public preference for cat management [7], [15]–[18]. Despite the aforementioned studies, a rigorous theoretical framework integrating multiple psychological constructs and stakeholder perspectives is lacking.

This research builds on previous studies to develop a theoretical model to understand stakeholder conflict. Despite a few notable exceptions, most of the studies related to outdoor cats have explored public perceptions [2], [14], [18]. This is problematic because stakeholders hold more polarized attitudes, beliefs and perceptions about outdoor cats and cat management, compared to the public [14]. Moreover, public participants are less concerned about this issue than stakeholders and therefore more likely to be influenced by the use of polarized language or biased framing [14]. Our model builds on existing research by evaluating attitudes, beliefs and perceptions among animal welfare advocates actively participating in TNR programs (referred to as “TNR supporters”) and members of the Audubon Society, representing the perspective of environmental advocates.

Previous studies have used biased language and/or framing to explore the issues associated with outdoor cats, by asking respondents to report perceptions of outdoor cats as a “problem for residents” [15], a “nuisance” or “pest”[19], [20]. Numerous studies in the wildlife management literature have recognized the potential benefits individuals perceive from animals (e.g., aesthetic enjoyment and recreational benefits) [4]. This is also true for outdoor cats; college students, caregivers and the public express widespread favorable feelings for these animals [17], [19], [21]. Therefore, our survey included questions about both negative and positive cat-related impacts.

TNR supporters expressed high levels of affection for outdoor cats and believed outdoor cats provide benefits to people by killing mice and pests and reducing the spread of disease [14]. Affect, or feelings have been identified as an important determinant of judgment [22], decision making [22], [23], perceptions of animals [24], and perceptions of risk and benefit [25]. Therefore, we measured the influence of attitudes toward cats (i.e., affection) on stakeholder support for lethal management.

We developed a parsimonious model of the cognitive antecedents of stakeholder conflict over animal management, and used outdoor cats to explore the utility of this model. Our model is based on the framework of the cognitive hierarchy [26] and uses previously validated multi-item scales [17]. The cognitive hierarchy suggests a hierarchical relationship between cognitions [27]–[30]. Each step in the proposed hierarchy builds on the next; values form the foundation and influence beliefs, which in turn influence attitudes and behavioral intentions [27], [31], [32]. This framework is grounded in the value-belief-norm theory, an adaptation of Stern and Dietz’s original hypothesis that values predict environmental attitudes [33]. This model links environmental values with behaviors using individual beliefs as mediators [34]. The foundation for the value-belief-norm theory is the New Ecological Paradigm (NEP) scale [35]. NEP measures five components of an ecological worldview: the reality of limits to growth, antianthropocentrism, the fragility of nature’s balance, the rejection of exemptionalism, and the possibility of an ecocrisis [35]. Individuals who value the natural world, or express ecocentric worldviews, agree more strongly with the positive elements of the NEP scale while people with more anthropocentric values (or a more dominant social paradigm) agree more strongly with the negative items in the scale. Significant differences in NEP scores represent divergent environmental values among stakeholder groups [36]–[38]. In this study, we use NEP as a measure of environmental values and the foundation of our hierarchical model. The specificity hypothesis posits that the relationship between beliefs, attitudes and behavior is stronger when specific beliefs and specific attitudes predict specific behaviors [39]–[41]. If beliefs about the negative and positive impacts associated with outdoor cats are salient, then they will influence attitudes and management preference.

**Figure 1:**
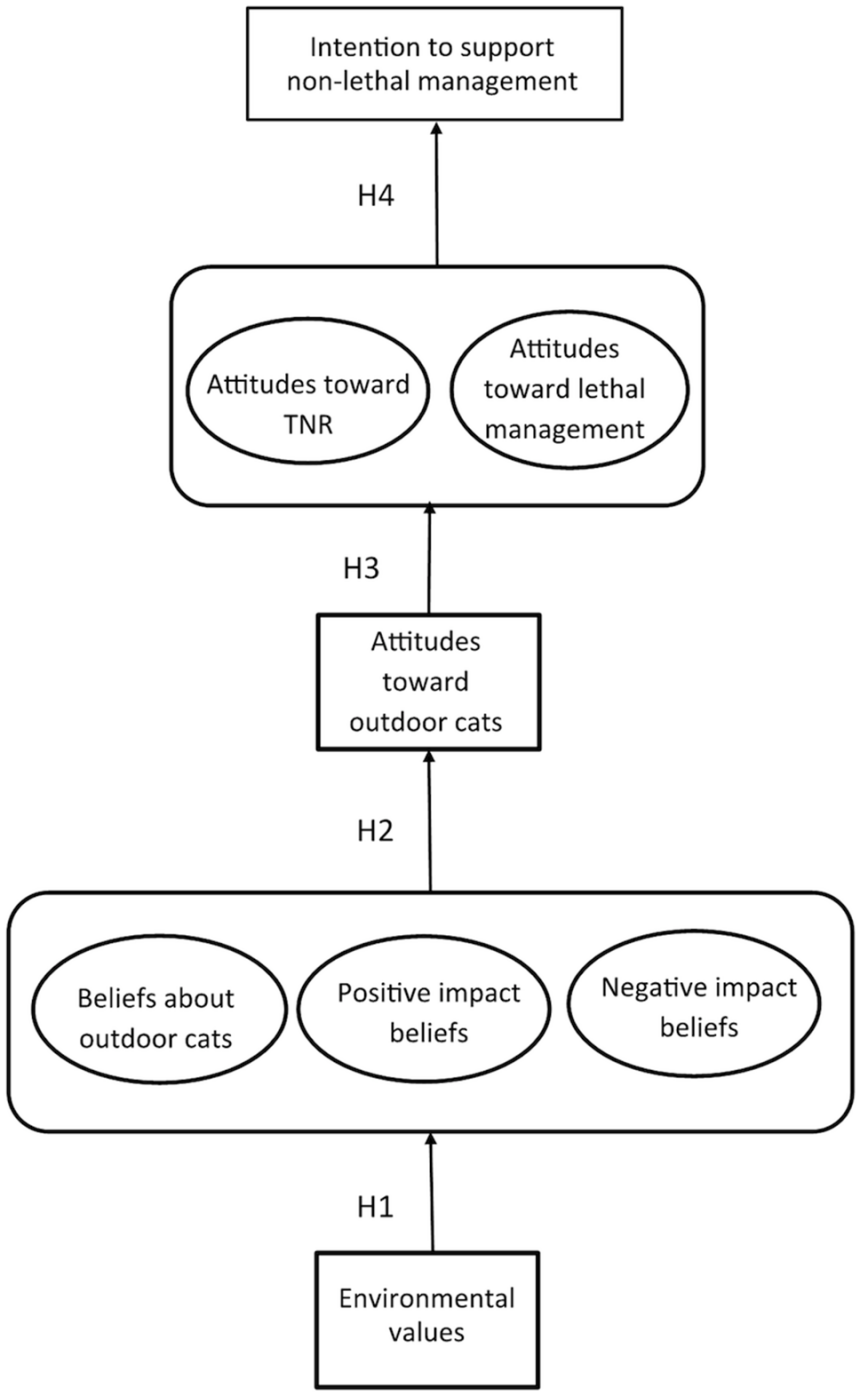
A theoretical model predicting intentions to support cat management. A theoretical model, based on the cognitive hierarchy, illustrating the hypothesized relationships between values, beliefs, attitudes and behavioral intentions. Each of the latent variables (represented by circles) is based on responses to a series of Likert-scale questions (Table 1).

**Figure 2:**
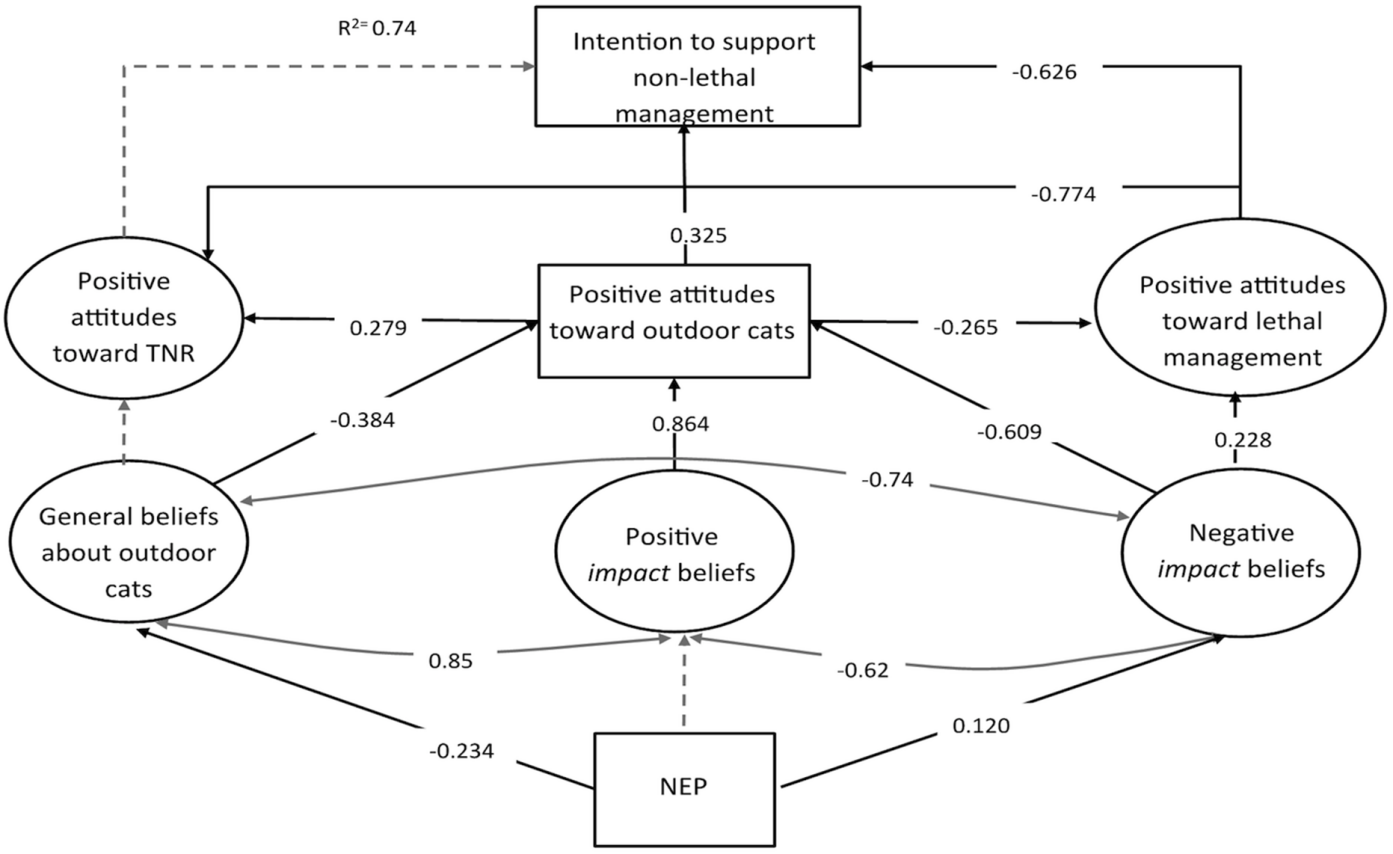
Path diagram for the best-fit structural equation model. In the model above, the latent variables are represented by circles; the rectangles represent additional single-item observed variables. Estimates reported for each of the paths represent standardized coefficients. Solid black lines indicate significant direct effects (p<0.05); insignificant paths are indicated with dashed lines. Correlations between the belief items are indicated by gray curved lines with double arrows.

Using a structural equation model, we (1) identified a model of the cognitive factors influencing intention to support cat non-lethal management (i.e., TNR and placement in a long-term no-kill shelter) (Figure 1); (2) tested the specificity hypothesis; (3) identified interactions between cognitive variables (i.e., values, beliefs, and attitudes) and (4) measured both direct and indirect effects on management support.

The model tested four hypotheses.

H1: Environmental values (NEP) will predict general and specific beliefs. (a) Respondents with ecocentric values (positive NEP score) will express fewer positive impacts about outdoor cats than individuals with dominant values. (b) Respondents with ecocentric values will perceive significantly more negative impacts from cats than individuals with dominant values.
H2: Beliefs will influence attitudes toward outdoor cats. (a) Respondents who believe cats have the right to live outdoors will express more positive attitudes about outdoors cats. (b) Respondents who agreed with positive cat impact beliefs will express more positive attitudes about outdoor cats. (c) Respondents who express greater agreement with negative impact beliefs will express more negative attitudes about outdoor cats.
H3: General attitudes will mediate the relationship between beliefs and specific attitudes. (a) Respondents with positive attitudes about outdoor cats will report more positive attitudes toward TNR. (b) Respondents with positive attitudes about outdoor cats will perceive lethal management as less humane.
H4: Both general and specific attitudes will influence behavioral intentions. (a) Respondents with positive attitudes about TNR will express greater support for non-lethal management than respondents with negative attitudes about TNR. (b) Respondents who view lethal management as humane will express less support for non-lethal management.

## Methods

### Ethics Statement

The Institutional Review Board (IRB) for the University of Florida approved this study. The IRB waived the need for written informed consent for the mail surveys because this research involved minimal risk for the participants. We collected written informed consent documents for the focus group research. All identifying information was removed prior to data analysis.

### Survey Instrument

Methods have been reported elsewhere [14]. Briefly, stakeholder groups, with divergent positions on support for cat management (TNR supporters and Audubon Society members), in four counties, representing both North and South Florida, were identified and recruited for this research. Participant names were randomly drawn from a sample of members/participants from existing TNR groups and Audubon Society chapters (TNR n = 800 and Audubon n = 796). The self-administered survey was distributed through the U.S. Postal Service.

Survey questions were pre-tested for validity and reliability using focus groups and in-person survey methods [17]. In focus groups, the term “outdoor cats” was identified as the most neutral and easy to understand term for describing free-roaming domestic cats. For all questions we asked respondents to report answers about outdoor cats not owned by them (File S1). In this study, we focus on survey responses to questions about environmental values, general beliefs about cats and specific beliefs about cat impacts, general attitudes toward outdoor cats, specific attitudes toward TNR and lethal management, and intentions to support management.

A multivariate model allowed us to simultaneously measure relationships between numerous exogenous and endogenous variables. Our proposed model included five continuous factors or latent variables (i.e., not directly observable) (1) beliefs about outdoor cats, (2) negative and (3) positive beliefs about cat impacts, and specific attitude constructs (4) attitudes toward TNR and (5) attitudes about lethal management (Figure 1; ovals), two continuous, observed variables (attitudes toward outdoor cats and environment values), and one observed, categorical factor indicator or dependent variable (intention to support non-lethal management) (Figure 1; boxes).

### Variables

#### Environmental values

Environmental values were measured using the 15-item updated New Ecological Paradigm (NEP) scale (Table 1) [26]. Items were collapsed into a 5-point Likert scale (1 = strongly disagree and 5 = strongly agree). NEP statements with a negative loading were reverse coded. Previous research has confirmed that the NEP items load on one factor and produce a single reliable measure [26]. After confirming the reliability of our measure, we followed the recommendation of Dunlap et al. 2000, and combined the NEP Scale into one composite variable (Table 1).

**Table 1:** Reliability and confirmatory factor analysis in the final structural equation model.

Survey item	Factorloading^1^	Cronbach’sα
Environmental Values (NEP)		0.86
We are approaching the limit of the number of people that the earth can support	0.38	
Humans have the right to modify the natural environment to suit their needs^2^	0.74	
When humans interfere with nature it often produces disastrous consequences	0.66	
Human ingenuity will insure that we do NOT make the earth unlivable^2^	0.40	
Humans are severely abusing the environment	0.46	
The earth has plenty of natural resources if we just learn how to develop them^2^	0.52	
Plants and animals have as much right as humans to exist	0.57	
The balance of nature is strong enough to cope with the impacts of modern industrial nations^2^	0.48	
Despite our social abilities humans are still subject to the laws of nature	0.44	
The so-called “ecological crisis” facing humankind has been greatly exaggerated^2^	0.61	
The earth is like a spaceship with very limited room and resources	0.35	
Humans were meant to rule over the rest of nature^2^	0.71	
The balance of nature is very delicate and easily upset	0.69	
Humans will eventually learn enough about how nature works to be able to control it^2^	0.52	
If things continue on their present course, we will soon experience a major ecological catastrophe	0.57	
General beliefs about outdoor cats		0.90
Cats deserve to be outdoors and free like other animals	0.76	
Wildlife and cats should have equal access to the outdoors	0.75	
Outdoor cats should have the right to hunt	0.77	
Outdoor cats live happy and healthy lives	0.74	
Outdoor cats are a problem in Florida^2^	0.69	
Perceived negative impacts beliefs		0.88
The use of my yard as a litter box by outdoor cats is a nuisance	0.83	
Outdoor cats spread diseases to people	0.74	
Outdoor cats make loud calls and noises	0.66	
Outdoor cats can spread diseases to owned pets	0.77	
Outdoor cats compete with wildlife for food	0.72	
Outdoor cats pose a significant risk to wildlife	0.73	
Perceived positive impact beliefs associated with outdoor cats		0.84
Outdoor cats kill mice and pests	0.51	
By killing pests, outdoor cats reduce the spread of disease	0.72	
Outdoor cats provide me with companionship	0.76	
Outdoor cats improve my quality of life	0.84	
Attitudes toward TNR		0.86
I support programs to trap-neuter and return outdoor cats	0.79	
Trap-neuter and return programs are a good way to manage outdoor cats	0.75	
I support using tax dollars for low-cost spay-neuter and return programs	0.59	
Attitudes toward lethal management		0.77
Placement in a short-term shelter followed by euthanasia	0.90	
Veterinary induced euthanasia	0.84	
Shooting	0.50	
Poisoned baits	0.42	

1 Factor loadings were standardized and were all significant at *p*<0.001. Factor loadings suggest acceptable correlations between each of the multi-item variables. Moreover, factor loadings suggest 5 latent factors (general beliefs, negative impact beliefs, positive impact beliefs, attitudes toward TNR, and attitudes toward lethal management. Environmental values were combined into one composite, continuous observed variable).

2 Items were reverse coded.

#### Beliefs

Six items representing general beliefs about outdoor cats (not necessarily objective facts (e.g., Vaske 2008) and 12 beliefs about cat impacts (8 negative and 4 positive belief items) were measured on a 7-point scale (1 = strongly disagree and 7 = strongly agree) (Table 1).

#### Attitudes

Three items measured attitudes toward TNR on a 7-point scale (1 = strongly disagree and 7 = strongly agree); high scores represent positive attitudes toward TNR (Table 1). Attitudes toward four lethal management approaches were measured (i.e., shelter euthanasia, veterinary euthanasia, shooting, and poisoning) on a 7-point scale (1 = inhumane and 7 = very humane; Table 1). Attitudes toward outdoor cats were measured by rating the statement, “What are your feelings about outdoor cats” on a 7-point scale (1 = unfavorable and 7 = favorable).

#### Intention to support non-lethal management

Support for management was used as a proxy for behavioral intention. To operationalize this categorical factor indicator, participants were asked to choose between four management choices and select a single preferred management approach. The four management choices listed are the most common methods currently used to manage outdoor cats. Choices included TNR, placement in a long-term, no kill sanctuary, trap and euthanize and no management. Items were then collapsed into a binary measure (0 = lethal methods, including trap and euthanize; and 1 = non-lethal methods, including the other three management choices).

For each survey participant, four socio-demographic items were measured: cat ownership, cat feeding, gender, and stakeholder group membership. We used IBM SPSS 20.0 and AMOS 20.0 for Windows for the initial analysis. Our final model was estimated using the WLSM estimator (weighted least squares parameter estimate) in Mplus Version 7.11 and employed a general analysis type [42]. Variance explained was reported as the squared multiple correlation coefficient (R^2^) [43].

## Results

### Tests for Sample Bias

We tested non-response bias by evaluating the differences between early and late survey respondents [44]. First round respondents (n = 620) and late respondents (n = 161) were compared on 10 questions from the survey and their responses did not differ significantly.

### Characteristics of Respondents

A total of 781 surveys were returned; Audubon members (n = 416, response rate = 52%) and TNR supporters (n = 365, response rate = 46%). Most respondents were female (78%), cat owners (63%), and did not feed outdoor cats (67%). We believe the large number of female responses reflects actual differences in the demographic composition of these stakeholder groups. Additionally, we were not interested in directly extrapolating the results of this model to the general public. Therefore the model presented here is based on unweighted data.

### Preliminary Analysis

A confirmatory factor analysis was conducted for the 15-item New Ecological Paradigm (NEP) scale (Table 1). Consistent with previous research findings, all NEP items loaded on one factor and produced a single reliable measure **(** Table 1; Eigenvalue = 5.23). Therefore we followed the recommendation of Dunlap et al. 2000, and combined the NEP Scale into one composite variable with a range of 1.8–5 (higher scores represent stronger agreement with NEP and more ecocentric values; Table 1). The multi-item scales developed for this study were previously tested for reliability and reported in Wald, Jacobson and Levy, 2013 and included 12 items. In this study, two items were removed from this analysis because a large number of missing responses reduced item reliability. Each scale was tested for reliability with Cronbach’s α>0.65 considered acceptable [45], [46]. One item, “cats should be kept indoors as pets,” reduced scale reliability and was removed from the general beliefs scale, resulting in a final 5-item scale (Table 1). The reliability of the multi-item scales was acceptable, Cronbach’s α>0.80 (Table 1).

As part of the model estimation, confirmatory factor analyses was conducted for each of our multi-item latent (unobserved) variables: (1) beliefs about outdoor cats, (2) positive impact beliefs, (3) negative impact beliefs, (4) attitudes toward TNR, and (5) attitudes toward lethal management. All items loaded acceptably on the multi-item latent variables (Table 1).

### Model Results

We fit the observed data to the proposed model. Responses with missing data were removed from the model (n = 298). The Comparative Fit Index (CFI), Root Mean Square Error of Approximation (RMSEA), and the theoretical meaning of the model were used to assess model fit. The CFI has a range of zero to 1.00, and values >0.90 are considered acceptable [47]. RMSEA values <0.08 are considered acceptable and <0.05 are considered good [47]. We used post hoc modification indices to identify additional parameters that enhanced model fit, but made adjustments supported by existing theory.

The initial model was not a good fit to the data (CFI = 0.56). We therefore adjusted the model post hoc based on modification indices and standardized factor loadings. The revised model was nested within the original model. We allowed error terms to correlate between the positive, negative and general belief items, two attitudes toward lethal management items (shooting and poisoning cats), two items in the positive impact beliefs factor (cats reduce disease and kill pest species), and the beliefs about cats factor (deserve to live outdoors and should have access to the outdoors equal to wildlife). In addition to improving the model, the correlated items also made theoretical sense. Negative beliefs about risks or impacts decrease as positive beliefs about risks increase [23]. Correlations indicated a significant and inverse relationship between negative impact beliefs and general beliefs about outdoor cats (–0.74, *p*<0.001) and positive impact beliefs (–0.62, *p*<0.001). There was a positive and direct correlation between positive impact beliefs and general beliefs about outdoor cats (0.85, *p*<0.001). The perceptions of the humaneness of shooting and poisoning cats were correlated (0.56, *p*<0.001), as were beliefs about cats reducing disease and killing pest species (0.57, *p*<0.001) and support for cats living outdoors and having access equal to wildlife (0.85, *p*<0.001).

In the final model, gender was not a significant predictor. Feeding cats directly influenced positive impact beliefs (β = 0.338, p<0.001), negative impact beliefs (β = –0.135, p<0.05) and general beliefs about outdoor cats (β = 0.217, p<0.001). Cat ownership influenced positive impact beliefs (β = 0.363, p<0.001), negative impact beliefs (β = –0.135, p<0.001), beliefs about outdoor cats (β = 0.296, p<0.001), and attitudes toward lethal management (β = –0.221, p<0.001). There was a significant relationship between group membership and negative impact beliefs (β = –0.144, p<0.001), and attitudes toward TNR (β = −0.175, p<0.05). There was a moderate to low level of correlation (<0.42) between the socio-demographic variables. Therefore, demographic variables were uncorrelated in the final model.

The revised model exhibited an acceptable fit (CFI: 0.94, RMSEA: 0.06). To compare model fit, we computed the Satorra-Bentler scaled (mean-adjusted) chi-square difference test [48]. The final model represented a significant improvement over the initial model (Table 2). Paths with insignificant relationships were trimmed from the final model diagram (Figure 2).

**Table 2:** Test statistics for hypothesized multivariate model.

Model	χ^2^	*df*	CFI^a^	RMSEA^b^	Scaling correction factor^c^	Δ*df*	S-B scaled chi-square dif (TRd)^d^
1. Initial	2965	329	0.56	0.164	0.55		
2. Final	738	349	0.94	0.062	0.46	20	1154**

a Confirmatory fit index.

b Root mean square error of approximation.

c Scaling correction factor provided by Mplus output for WLSM.

d Satorra-Bentler chi-square difference test result.

**p<0.005.

All causal paths, except general beliefs to positive attitudes, had signs in the expected direction and results supported almost all of the hypothesized relationships (Figure 2). Environmental values did not predict positive impact beliefs (H1a). Respondents with ecocentric values held greater negative impact beliefs about cats (H1b). The relationship between general beliefs about outdoor cats and positive attitudes toward outdoor cats was negative (H2a). Positive impact beliefs increased positive attitudes toward cats, while negative impact beliefs decreased positive attitudes (H2b, H2c). Neither positive or negative impact beliefs directly predicted attitudes toward TNR, but negative impact beliefs directly increased perceived humaneness of lethal management. Positive attitudes about outdoor cats increased support for TNR (H3a), decreased perceived humaneness of lethal management (H3b) and increased intention to support non-lethal management. Support for TNR decreased with increased perceptions of the humaneness of lethal management. Positive attitudes toward TNR and intentions to support non-lethal management were highly correlated (0.85), but there was no significant direct effect (H4a). Perceived humaneness of lethal management significantly reduced intentions to support non-lethal management (H4b). Attitudes toward lethal management had the largest total standardized effect on non-lethal management support and positive attitudes toward TNR (Figure 2). Positive impact beliefs had the largest total standardized effect on positive attitudes toward outdoor cats (Figure 2). The combine latent factors and measured variables explained 74% of the observed variance in intention to support non-lethal management.

Significant indirect effects were estimated using the Sobel test, also described as the Delta Method [49]–[51]. An understanding of indirect effects helps identify the underlying psychological antecedents of management support. An indirect effect occurs when the relationship, or total effect, between a predictor and outcome variable significantly changes with the addition of an intermediate variable (e.g., A causes B, and B causes C)[52].

The total indirect effect of environmental values on support for non-lethal management was not significant, but the specific path from values to lethal management through general beliefs and attitudes toward outdoor cats was significant (β = 0.03, p<0.05). Positive impact beliefs and negative impact beliefs had significant total indirect effects on support for non-lethal management (Table 3). In addition, specific indirect effects of beliefs on support for non-lethal management, mediated by general attitudes toward outdoor cats and specific attitudes toward lethal management, were significant for both negative impact beliefs (β = –0.10, p<0.05) and positive impact beliefs (β = 0.14, p<0.05). Test results indicated that negative and positive impact beliefs, as well as general attitudes toward cats and specific attitudes toward lethal management had significant indirect effects on support for non-lethal management (Table 3). Negative impact beliefs had the largest total indirect effect on management support; followed closely by positive impact beliefs (Table 3). These results support our hypotheses that beliefs influence attitudes toward outdoor cats (H2), general attitudes mediate the relationship between beliefs and specific attitudes (H3), and both general and specific attitudes influence behavioral intentions.

**Table 3:** Standardized total indirect effects of independent variables on support for non-lethal cat management.

Model	NEP	General Beliefs	Neg Impact Beliefs	Pos Impact Beliefs^a^	General Attitudes	
Standardized total indirect effects on management support						
Final	–0.009(–0.016)^b^	–0.188(–0.222)	–0.441(–0.520)**	0.423(0.500)*	0.166(0.091)*	

a Total effect  =  indirect effect + direct effect.

b StdYX estimate (Std estimate).

*p<0.05;

**p<0.001.

## Discussion

Using the cognitive hierarchy framework allowed us to observe hierarchical relationships among cognitions that could have been missed using multiple linear or logistic regression analyses, as has been previously applied [7], [15], [16]. Our results confirm that the reasons for individual management preference are multifaceted and influenced by multivariate relationships between cognitive factors [53]. Our results add to the growing evidence that specific attitudes and general attitudes are important predictors of behavioral intentions. Previous research has identified socio-demographic variables [54], [55], value orientations [15], [16], and attitudes about cat management [7], [17] as key variables for predicting public support for management preference. While we agree that these variables are important, our model was a better fit to the data when gender was removed and none of the demographic variables directly predicted general attitudes toward cats or behavioral intention to support management. It is possible that socio-demographic variables are more important drivers of public preference while attitudes and strongly held beliefs are more predictive for active stakeholders. This research provides valuable information about the variables influencing stakeholder support for management, specifically the use of lethal methods for controlling the outdoor cat population.

### Cognitive Hierarchy Framework

We included demographic variables in our model because previous research reports significant relationships between demographic variables and management preferences [15], [54], [55]. However, researchers have also found that when demographic, experiential variables and knowledge are compared to attitudes, as predictors of tolerance for cats and management preference, “attitudes toward cat management” has the largest effect on preference for cat management [7], [17]. Our multivariate model confirmed these results by demonstrating significant direct effects of cat ownership and feeding on beliefs about outdoor cats and cat-related impacts, but not on non-lethal management support.

This research enhances our understanding of stakeholder preferences by considering how both specific attitudes toward TNR and attitudes about the humaneness of lethal management influence management support independently. As hypothesized, results supported the relationships advanced by the cognitive hierarchy and provided support for the specificity hypothesis, which posits that the relationship between beliefs, attitudes, and behaviors is stronger in cases where the beliefs address specific situations or issues [28]–[30], [56]. In our model, general attitudes toward cats mediated the relationship between negative and positive impact beliefs and specific attitudes for cats, similar to the role that general attitudes play in the relationship between values and specific wildlife protection attitudes [57]. The important and significantly predictive relationships between negative and positive impact beliefs, general attitudes toward outdoor cats and perceptions of the humaneness of lethal management suggest that in the case of outdoor cats, attitudes about whether the proposed management method is appropriate and/or humane may be more strongly related to attitudes about and preferences for non-lethal management than general attitudes or beliefs about the referent species. While positive attitudes toward TNR were not a significant predictor of non-lethal management support, they were strongly correlated. It is possible this finding is the result of combining our non-lethal management types into one binary predictor or underlying correlations between the attitude items.

Other models and approaches have highlighted the hierarchical relationship between values, attitudes, and behaviors and the importance of attitude specificity. In this study, ecocentric values contributed to both increased negative impact beliefs and decreased positive general beliefs about outdoor cats. Our findings support the applicability of a model based on the cognitive hierarchy with environmental values as a basis for beliefs about animals and animal impacts, which predict attitudes, that, in-turn influence behavioral intentions. Positive beliefs about cats were not directly predicted by environmental values suggesting that these beliefs may be influenced by a different set of underlying values.

Our sample included a small number of male respondents. The sample was not weighted because we believed this reflected actual differences in the demographic composition of these stakeholder groups. Moreover, including gender did not enhance our model. However, future research should explore the possible interaction between gender, cognitive variables and management support for outdoor cats. Because our model was evaluated with a sample of active stakeholders, it is currently limited in its generalizability to the public, who may or may not posses the same level of interest or knowledge about the management of outdoor cats. However, previous research findings have suggested that many of the variables included in this model are important predictors of management preference for the public, and that attitude-based models are a better predictor of management preference than models focused on demographic variables, experiences and knowledge [7], [14], [17]. Future research should explore the applicability of this model to a sample of the general public.

### Model Contribution

Animal management efforts are influenced by stakeholder perceptions and support [58]. Reliable information about stakeholder attitudes toward management and support for management is a crucial step in minimizing conflict over the lethal management of animals. Our model confirmed that both negative (i.e., environmental risk) and positive (i.e., benefits) impact beliefs significantly influence general and specific attitudes about outdoor cats. Moreover, our results confirm previous findings suggesting that individuals may simultaneously hold both negative and positive beliefs [59]. Stakeholders can believe that cats provide both negative and positive impacts and simultaneously express strong and affectionate feelings toward outdoor cats, but express concerns about cat’s spending time outdoors. It is important to acknowledge this complexity and move away from a characterization of the stakeholders engaged in groups that support or oppose TNR as one-sided. We hope that our model, which demonstrates the importance of attitudes about lethal and non-lethal management methods, can inform efforts to engage stakeholders, reduce conflict over animal management, and engender support for policies aimed at managing the outdoor cat population.

Few studies have measured the potential benefits cat owners, cat feeders, and colony managers perceive from outdoor cats, or have evaluated the role these benefits play in attenuating perceived risks or influencing tolerance for outdoor cats and management support [17]. The perceived benefits associated with outdoor cats (e.g., companionship) are not as apparent, for instance, as the negative impacts (e.g., predation, disease) often highlighted in the media. Previously developed univariate models have illustrated that ecological risk perceptions (i.e., the perceived threat to wildlife and ecological systems) influence public and stakeholder attitudes toward the management of cats [2], [7]. The relationship between positive impact beliefs and positive attitudes and negative impact beliefs and negative attitudes supports the theory that individuals strive for cognitive consistency because possessing dissonant beliefs about specific objects or events is uncomfortable [60]. The observed negative correlation between negative and positive impact beliefs may be attributed to the previously documented inverse relationship between perceived risks and benefits [25]. The observed direct relationship between positive impact beliefs and positive attitudes toward cats is consistent with previously observed relationships between affect and perceived risks and benefits [23]. Given these relationships, and the mediating effect of general attitudes on general beliefs and specific attitudes, strongly held perceived benefits or affection for cats may attenuate risk perceptions [23], [61]. Our findings suggest that combining negative and positive beliefs or ignoring positive beliefs completely could lead managers to mischaracterize stakeholder support/opposition to animal management methods.

Previous research suggests that stakeholders have strongly held beliefs about whether cats pose a risk to wildlife [2], [14]. However, few studies had directly measured these beliefs among stakeholders or evaluated their influence on management preferences. Our model suggests that differing beliefs about the positive and negative impacts of cats on wildlife, people, and the environment play an important role in influencing attitudes toward lethal management and support for non-lethal management (Figure 2). A number of the belief items included in the survey were drawn from peer-reviewed scientific literature about cat behavior and a number were drawn from opinions expressed by stakeholders in focus groups. Whether the beliefs were grounded in scientific evidence or based on opinions and evidence presented by more informal personal networks, they directly influenced participant attitudes toward management. This has important implications for educational campaigns aimed at generating stakeholder support for cat management methods.

### Implications for Animal Management

Knowledge of the values underlying attitudes and management support contributes to existing theoretical models of human behavior and can reduce conflict over environmental attitudes and natural resource management [62]. Preference for wildlife management is influenced by perceived management effectiveness, animal suffering, environmental impacts, problem severity [63], and beliefs about the outcomes of lethal control [53]. Support for lethal management for white-tailed deer (*Odocoileus virginianus*), beaver (*Castor canadensis*) and Canada geese (*Branta canadensis*) was related to beliefs about animals as a nuisance [64]. The acceptability of lethal wildlife management increased as the severity of the impacts to people increased [65]. Negative experiences with outdoor cats (e.g., problems with cats on property, killing birds or small mammals or scaring birds from birdfeeders) significantly reduced public support for TNR (p<0.001) [15]. In this study, individuals who perceived negative impacts to wildlife from outdoor cats and held negative attitudes about outdoor cats were more likely to perceive the use of lethal management methods for cats as humane. Previous focus groups, conducted with stakeholders involved in the management of feral cats and deer, also found that ethical judgments about management influenced stakeholder attitudes toward management [18]. In our model, perceived humaneness of lethal management had the largest direct effect on management support and attitudes toward TNR and suggests that attitudes toward lethal management are also critical influences on preference for the management of domesticated animals, such as outdoor cats. We would encourage managers to explore creative management techniques that build collaboration between stakeholder groups by identifying management methods that are perceived as humane and effective by both groups. This may provide an area of agreement between stakeholders who otherwise disagree about risks from predation or benefits from companionship. Thus a focus on potential areas of agreement about outdoor cats might also enhance collaboration between animal welfare advocates and environmental advocates. In addition to reducing stakeholder conflict over the management of outdoor cats, our findings should also inform efforts to manage other non-native species, such as mute swans (*Cygnus olor)* in Maryland [66], or feral pigs (*Sus scrofa*) in Hawaii [67], that have been stymied by stakeholder opposition to proposed management strategies.

Our study also has implications for public outreach programs. Both mass media and interpersonal communication are used to convey risk information, but previous studies have suggested “individuals select elements from media reports and use their own frame of reference to create understanding and meaning” [68, pg 1060]. A recent study found that respondents were less likely to correctly identify the solution to a mathematical problem if the correct answer contradicted their political beliefs [69]. Our model suggests that differing beliefs about cat-related impacts are a key predictor of attitudes and ultimately, support for cat management. Confirmation bias suggests that people with strongly held beliefs are less willing to modify their beliefs in the face of new and conflicting evidence [70]–[72]. While factual information can influence public opinion [59], [73], [74], it can also result in a “backfire effect” where the provision of objective information, aimed at correcting misperceptions, enhances support for a widespread misconception [75]. Therefore, providing individuals who strongly believe that cats do not pose a risk to wildlife with additional information about cats killing birds may not necessarily modify these beliefs, which are important predictors of attitudes toward cat management. Future research should explore whether risk or benefit based messages generate confirmation biases or “backfire” effects among stakeholders with strongly held beliefs about the use of lethal management methods.

## Supporting Information

File S1 **Full stakeholder survey.**
(PDF)
