# Supplementary material for: A Multivariate Model of Stakeholder Preference for Lethal Cat Management
Source: PLoS One. 2014 Apr 15;9(4):e93118. doi: 10.1371/journal.pone.0093118 (PMC3988042; doi:10.1371/journal.pone.0093118)
Supplement: File S1 — Full stakeholder survey. (PDF) [file pone.0093118.s001.pdf]

A Survey of Florida Residents

A study of resident opinions about outdoor cats

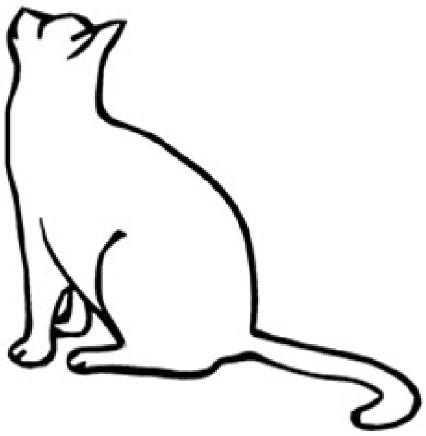

Thank you for sharing your opinions about outdoor cats in your community. This survey should take only a few minutes to complete. Your opinions are very important to us.

Please mail your completed survey in the enclosed envelope to:

Dara Wald
University of Florida
PO Box 110430
Gainesville, FL 32611-0430
Phone: (352) 392-8372

YOUR EXPERIENCE

There are many terms to describe domestic cats, including owned cats that stay inside or spend some time outside, friendly strays, and unapproachable feral cats. In this survey, any cats that spend time outside will be referred to as **outdoor cats**.

The following questions refer to your experiences with outdoor cats **not owned** by you.

1. Indicate which, if any, of the following types of interactions you have personally experienced with outdoor cats. *(Please check [√] **all** that apply)*

	Yes	No
a. Observed an outdoor cat in my neighborhood	☐	☐
b. Observed an outdoor cat in my yard	☐	☐
c. Observed an outdoor cat hunting	☐	☐
d. Observed an outdoor cat scaring birds at a bird feeder	☐	☐
e. Had a pet threatened or attacked by an outdoor cat	☐	☐
f. Given food to an outdoor cat	☐	☐
g. Have heard or read about problems with outdoor cats	☐	☐
h. Have heard or read about benefits from outdoor cats	☐	☐
i. Pet an outdoor cat	☐	☐
j. Have been injured by an outdoor cat	☐	☐
k. Adopted an outdoor cat	☐	☐
l. Trapped an outdoor cat	☐	☐
m. Seen cats killed on the road	☐	☐
n. Had outdoor cats disturbing my garden	☐	☐

p. Other types of experiences: ____

2. How often do you see outdoor cats (not owned by you) in your neighborhood?
*(Please check [√] only **one** of the following statements)*

☐ Don't know
☐ Never
☐ Rarely
☐ Occasionally
☐ Frequently
☐ Everyday

3. How has the outdoor cat population changed in your neighborhood in the last five years?
(Please circle the number that best represents your response to each statement)

Decrease greatly						Increase greatly	Don't know
1	2	3	4	5	6	7	DK

YOUR OPINIONS

The following questions are designed to measure your opinions about outdoor cats.

4a. Which category best describes your feelings about outdoor cats?
(Please circle the number that best represents your response to the statement above)

Unfavorable feeling						Favorable feeling	Don't know
1	2	3	4	5	6	7	DK

4b. Which category best describes your feelings about domestic cats in general?
(Please circle the number that best represents your response to the statement above)

Hate						Love	Don't know
1	2	3	4	5	6	7	DK

5. People have many different feelings towards outdoor cats. To what extent do you agree or disagree with each of the following statements?
(Please circle the number that best represents your response to each statement)

Statement	Strongly disagree						Strongly agree	Don't know
a. Cats deserve to be outdoors and free like other animals	1	2	3	4	5	6	7	DK
b. Wildlife and cats should have equal access to the outdoors	1	2	3	4	5	6	7	DK
c. Cats should be kept indoors as pets	1	2	3	4	5	6	7	DK
d. More people should take responsibility for outdoor cats	1	2	3	4	5	6	7	DK
e. I want to help outdoor cats	1	2	3	4	5	6	7	DK
f. I worry about the health of outdoor cats	1	2	3	4	5	6	7	DK
g. I like seeing outdoor cats	1	2	3	4	5	6	7	DK
h. Outdoor cats are a problem in Florida	1	2	3	4	5	6	7	DK

Question 5 continued…
People have many different feelings toward outdoor cats. To what extent do you agree or disagree with each of the following statements?
(Please circle the number that best represents your response to each statement)

Statement	Strongly disagree						Strongly agree	Don't know
i. Outdoor cats should have the right to hunt	1	2	3	4	5	6	7	DK
j. Outdoor cats should be vaccinated against rabies	1	2	3	4	5	6	7	DK
k. Outdoor cats should have access to food	1	2	3	4	5	6	7	DK
l. Outdoor cats should have access to shelter	1	2	3	4	5	6	7	DK
m. Outdoor cats live happy and healthy lives	1	2	3	4	5	6	7	DK
n. Most outdoor cats are able to find their way home on their own	1	2	3	4	5	6	7	DK
o. Most outdoor cats are able to survive without human help	1	2	3	4	5	6	7	DK
p. I worry about the welfare of outdoor cats	1	2	3	4	5	6	7	DK

THE OUTDOOR CAT POPULATION

The following section asks for your opinion about the outdoor cat population in your neighborhood

6a. In your neighborhood, there are
(Please circle the number that best represents your response to each statement)

Too many outdoor cats				Too few outdoor cats	Don't know
1	2	3	4	5	DK

6b. What you would like to happen to the outdoor cat population in your neighborhood?
(Please circle the number that best represents your response to each statement)

Decrease greatly						Increase greatly	Don't know
1	2	3	4	5	6	7	DK

RISKS and BENEFITS

The following questions are designed to measure your perceptions of the risks and benefits associated with outdoor cats.

7a. People have many perceptions of outdoor cats. To what extent do you agree or disagree with each of the following statements?
(Please circle the number that best represents your response to each statement)

Statement	Strongly disagree						Strongly agree	Don't know
a. Outdoor cats are at risk of being killed or injured by people	1	2	3	4	5	6	7	DK
b. Outdoor cats are at risk of contracting diseases, such as rabies	1	2	3	4	5	6	7	DK
c. Outdoor cats are at risk of injury from other free-roaming pets	1	2	3	4	5	6	7	DK
d. Outdoor cats kill mice and pests	1	2	3	4	5	6	7	DK
e. By killing pests, outdoor cats reduce the spread of diseases	1	2	3	4	5	6	7	DK
f. Outdoor cats provide me with companionship	1	2	3	4	5	6	7	DK
g. Outdoor cats improve my quality of life	1	2	3	4	5	6	7	DK
h. The use of my yard as a litter box by outdoor cats is a nuisance	1	2	3	4	5	6	7	DK
i. Outdoor cats spread diseases to people	1	2	3	4	5	6	7	DK
j. Outdoor cats make loud calls and noises	1	2	3	4	5	6	7	DK
k. Outdoor cats can spread diseases to owned pets	1	2	3	4	5	6	7	DK
l. Outdoor cats compete with wildlife for food	1	2	3	4	5	6	7	DK
m. Outdoor cats spread diseases to wildlife	1	2	3	4	5	6	7	DK
n. Outdoor cats kill wildlife	1	2	3	4	5	6	7	DK
o. Outdoor cats pose a significant risk to wildlife	1	2	3	4	5	6	7	DK

7b. How important are the following issues to you?
(Please circle the number that best represents your response to each statement)

Statement	Not at all important						Extremely important	Don't know
a. Outdoor cats are killed or injured by people	1	2	3	4	5	6	7	DK
b. Outdoor cats contract diseases, such as rabies	1	2	3	4	5	6	7	DK
c. Outdoor cats live shorter lives than indoor only cats	1	2	3	4	5	6	7	DK
d. Outdoor cats are injured or attacked by free-roaming pets	1	2	3	4	5	6	7	DK
e. Outdoor cats compete with wildlife for food	1	2	3	4	5	6	7	DK
f. Outdoor cats spread diseases to wildlife	1	2	3	4	5	6	7	DK
g. Outdoor cats kill wildlife	1	2	3	4	5	6	7	DK
h. Outdoor cats provide benefits to people	1	2	3	4	5	6	7	DK
i. Outdoor cats pose risks to people	1	2	3	4	5	6	7	DK

8. What level of risk do you think each item poses *to environmental health*?
(Please circle the number that best represents your opinion for each statement)

Statement	No risk						Serious risk	Don't know
a. Death of wildlife from diseases	1	2	3	4	5	6	7	DK
b. Death of wildlife from natural predators	1	2	3	4	5	6	7	DK
c. Death of wildlife from outdoor cats	1	2	3	4	5	6	7	DK
d. Wildlife poaching	1	2	3	4	5	6	7	DK
e. Wildlife deaths by car collision	1	2	3	4	5	6	7	DK
f. Climate change or global warming	1	2	3	4	5	6	7	DK
g. Habitat loss	1	2	3	4	5	6	7	DK
h. Logging or mining	1	2	3	4	5	6	7	DK
i. Pollutants in waterways	1	2	3	4	5	6	7	DK
j. The spread of non-native plants and animals	1	2	3	4	5	6	7	DK
k. Emissions from automobiles	1	2	3	4	5	6	7	DK
l. The illegal pet trade	1	2	3	4	5	6	7	DK

9a. How much risk do the following animals pose *to your health and safety?*
(Please circle the number that best represents your opinion for each statement)

Statement	No risk						Serious risk	Don't know
a. Raccoon	1	2	3	4	5	6	7	DK
b. Opossum	1	2	3	4	5	6	7	DK
c. Outdoor cat	1	2	3	4	5	6	7	DK
d. Free-roaming dog	1	2	3	4	5	6	7	DK
e. Bat	1	2	3	4	5	6	7	DK
f. Coyote	1	2	3	4	5	6	7	DK

9b. Which of the following animals is native or exotic.
(Please circle the number that best represents your opinion for each statement)

	Native	Exotic
a. Raccoon	1	2
b. Armadillo	1	2
c. Outdoor cat	1	2
d. White tailed deer	1	2
e. Bat	1	2
f. Coyote	1	2

YOUR PERCEPTIONS

The following questions are designed to help us understand your opinions about the interaction between outdoor cats, people, wildlife, and the environment.

10a. The effect of outdoor cats on me is
(Please circle the number that most closely represents your perception)

Negative						Positive	Don't know
1	2	3	4	5	6	7	DK
**Unacceptable**						**Acceptable**	**Don't know**
1	2	3	4	5	6	7	DK

10b. The effect of outdoor cats on native wildlife is
(Please circle the number that most closely represents your perception)

Negative						Positive	Don't know
1	2	3	4	5	6	7	DK
**Unacceptable**						**Acceptable**	**Don't know**
1	2	3	4	5	6	7	DK

10c. The effect of outdoor cats on the ecosystem is
(Please circle the number that most closely represents your perception)

Negative						Positive	Don't know
1	2	3	4	5	6	7	DK
**Unacceptable**						**Acceptable**	**Don't know**
1	2	3	4	5	6	7	DK

10d. What level of risk does predation by outdoor cats pose to wildlife?
(Please circle the number that most closely represents your perception)

Not serious						Extremely serious	Don't know
1	2	3	4	5	6	7	DK

10e. What level of risk do outdoor cats pose to the ecosystem?
(Please circle the number that most closely represents your perception)

Not serious						Extremely serious	Don't know
1	2	3	4	5	6	7	DK

10f. What level of risk do outdoor cats pose to me?
(Please circle the number that most closely represents your perception)

Not serious						Extremely serious	Don't know
1	2	3	4	5	6	7	DK

10g. Outdoor cats provide me with personal benefits
(please circle the number that most closely represents your opinion)

Strongly disagree						Strongly agree	Don't know
1	2	3	4	5	6	7	DK

MANAGEMENT

There are many different ways to manage and control outdoor cats. The questions below ask for your opinion about the management of these animals.

11a. Are you familiar with programs that Trap-Neuter and Return cats (Capture the animal, sterilize it and return the cat back to the area where it was found**?**

☐ Yes
☐ No

11b. To what extent do you agree or disagree with each of the following statements?
(Please circle the number that best represents your opinion for each statement)

Statement	Strongly disagree						Strongly agree	Don't know
a. I support programs to trap-neuter and return outdoor cats	1	2	3	4	5	6	7	DK
b. I support programs to trap and impound outdoor cats	1	2	3	4	5	6	7	DK
c. The outdoor cat population should be left alone	1	2	3	4	5	6	7	DK
d. I oppose any program that reduces the outdoor cat population	1	2	3	4	5	6	7	DK
e. Owners should be prohibited from allowing cats to roam outdoors	1	2	3	4	5	6	7	DK
f. Owners should be required to provide identification (tag or microchip) for their cats	1	2	3	4	5	6	7	DK
g. Local governments should be responsible for controlling outdoor cats	1	2	3	4	5	6	7	DK
h. Trap-neuter and return programs are a good way to manage outdoor cats	1	2	3	4	5	6	7	DK
i. Removal to an animal shelter is a good way to manage outdoor cats	1	2	3	4	5	6	7	DK
j. I support mandatory spay-neuter laws for cats	1	2	3	4	5	6	7	DK
k. I support laws requiring cats be vaccinated against rabies	1	2	3	4	5	6	7	DK
l. I support using tax dollars to fund animal control shelters	1	2	3	4	5	6	7	DK
m. I support using tax dollars for low-cost spay-neuter and return programs	1	2	3	4	5	6	7	DK
n. Local governments do a good job of managing outdoor cats	1	2	3	4	5	6	7	DK

12. Which of the following management actions do you prefer for managing unowned outdoor cats?
*(Please check [✓] only **one** of the following statements)*

☐	Trap-neuter, and return; Capture animal, sterilize and return the cat back to the outdoors
☐	Placement in a long-term, no kill sanctuary
☐	Trap and euthanize
☐	No management – Animal will remain outdoors without any help or assistance from people

13. In your opinion, how humane are the following methods for managing outdoor cats?
(Please circle the number that best represents your opinion)

Statement	Not humane						Very humane	Don't know
a. Placement in a long-term no-kill sanctuary	1	2	3	4	5	6	7	DK
b. Fertility control (such as spay/neuter)	1	2	3	4	5	6	7	DK
c. Trap-neuter, and return	1	2	3	4	5	6	7	DK
d. Doing nothing	1	2	3	4	5	6	7	DK
e. Placement in a short-term shelter followed by euthanasia	1	2	3	4	5	6	7	DK
f. Placement in a short-term shelter followed by adoption	1	2	3	4	5	6	7	DK
g. Veterinary induced euthanasia	1	2	3	4	5	6	7	DK
h. Shooting	1	2	3	4	5	6	7	DK
i. Poisoned baits	1	2	3	4	5	6	7	DK

14. If you saw an outdoor cat in your yard on a regular basis, you would
*(Please check [✓] only **one** of the following statements)*

☐	Take no action
☐	Contact a cat advocacy organization, such as the Humane Society
☐	Contact animal control to remove it
☐	Capture the animal and remove it to an animal shelter
☐	Try to find the owner
☐	Arrange for trap-neuter, and return
☐	Adopt it
☐	Feed it
☐	Don't Know

15a. Have you made attempts to control outdoor cats/kittens (not owned by you)?

☐ Yes
☐ No → ***If you answered NO, please skip to question number 16.***

*If you answered YES to the previous question, please continue **HERE**. If not, please continue to the next section.*

15b. Please indicate which, if any, of the following actions you have taken to control outdoor cats. *(Please check [√] **all** that apply)*

☐	Adopted an outdoor cat
☐	Found a home for an outdoor cat
☐	Paid to have a cat spayed or neutered
☐	Taken a cat to a long-term shelter or sanctuary (no kill)
☐	Taken a cat to a short-term shelter
☐	Contacted a trap-neuter and return organization
☐	Contacted animal control about nuisance cats
☐	None of the experiences above
	Other experiences ____________________

PEOPLE AND THE ENVIRONMENT

16. To what extent do you agree or disagree with each of the following statements? *(Please circle the number that best represents your response to each statement)*

Statement	Strongly disagree						Strongly agree	Don't know
a. We are approaching the limit of the number of people that the earth can support	1	2	3	4	5	6	7	DK
b. Humans have the right to modify the natural environment to suit their needs	1	2	3	4	5	6	7	DK
c. When humans interfere with nature it often produces disastrous consequences	1	2	3	4	5	6	7	DK
d. Human ingenuity will insure that we do NOT make the earth unlivable	1	2	3	4	5	6	7	DK
e. Humans are severely abusing the environment	1	2	3	4	5	6	7	DK
f. The earth has plenty of natural resources if we just learn how to develop them	1	2	3	4	5	6	7	DK
g. Plants and animals have as much right as humans to exist	1	2	3	4	5	6	7	DK

Question 16 continued…
To what extent do you agree or disagree with each of the following statements?
(Please circle the number that best represents your response to each statement)

Statement	Strongly disagree						Strongly agree	Don't know
h. The balance of nature is strong enough to cope with the impacts of modern industrial nations	1	2	3	4	5	6	7	DK
i. Despite our social abilities humans are still subject to the laws of nature	1	2	3	4	5	6	7	DK
j. The so-called "ecological crisis" facing humankind has been greatly exaggerated	1	2	3	4	5	6	7	DK
k. The earth is like a spaceship with very limited room and resources	1	2	3	4	5	6	7	DK
l. Humans were meant to rule over the rest of nature	1	2	3	4	5	6	7	DK
m. The balance of nature is very delicate and easily upset	1	2	3	4	5	6	7	DK
n. Humans will eventually learn enough about how nature works to be able to control it	1	2	3	4	5	6	7	DK
o. If things continue on their present course, we will soon experience a major ecological catastrophe	1	2	3	4	5	6	7	DK

BACKGROUND INFORMATION

17. How often do you participate in any of the following behaviors?
(Please check [√] the item that best represents your participation level for each activity)

	Daily	Weekly	Monthly	Once a year	Never
a. Recycle cans and bottles	[ ]	[ ]	[ ]	[ ]	[ ]
b. Use public transportation or a bicycle	[ ]	[ ]	[ ]	[ ]	[ ]
c. Purchase environmentally friendly products	[ ]	[ ]	[ ]	[ ]	[ ]
d. Pick up trash that is not your own	[ ]	[ ]	[ ]	[ ]	[ ]
e. Compost food scraps	[ ]	[ ]	[ ]	[ ]	[ ]
f. Vote for a candidate because he/she supports environmental issues	[ ]	[ ]	[ ]	[ ]	[ ]
g. Donate money to an environmental group	[ ]	[ ]	[ ]	[ ]	[ ]
h. Feed birds	[ ]	[ ]	[ ]	[ ]	[ ]
i. Donate money to an animal welfare group	[ ]	[ ]	[ ]	[ ]	[ ]
j. Vote for a candidate because he/she supports animal welfare issues	[ ]	[ ]	[ ]	[ ]	[ ]

18. Do you currently own cats?

☐ Yes
☐ No → *If you answered NO, please skip to question number **22.***

19. How many cats do you currently own?
(*Please check [✓] only **one** of the following statements)*

☐ 1
☐ 2
☐ 3
☐ 4
☐ More than 4

20. Are any of your cats allowed outdoors?

☐ Yes
☐ No

* *Cat owners* continue *HERE*. *If you do not own cats, please continue to question **22.***

21. Please indicate to what extent you agree or disagree with each of the following statements.
(Please circle the number that best represents your response to each statement)

Statement	Strongly disagree						Strongly agree	Don't know
a. I can control whether my cat(s) stay indoors	1	2	3	4	5	6	7	DK
b. I can control whether my cat(s) are spayed or neutered	1	2	3	4	5	6	7	DK
c. I can control whether my cat(s) are vaccinated against rabies	1	2	3	4	5	6	7	DK
d. I intend to keep my cat(s) indoors	1	2	3	4	5	6	7	DK
e. I intend to get my cat(s) spayed or neutered	1	2	3	4	5	6	7	DK
f. I intend to have my cat(s) vaccinated against rabies	1	2	3	4	5	6	7	DK

22a. Please fill in the following information.
*(Please check [√] **all** that apply)*

	I am a member of	I donate money to	I attend member meetings	I volunteer for
Organization that supports Trap-neuter and return	[ ]	[ ]	[ ]	[ ]
The Audubon Society	[ ]	[ ]	[ ]	[ ]
The Humane Society	[ ]	[ ]	[ ]	[ ]
Alley Cat Allies	[ ]	[ ]	[ ]	[ ]
American Bird Conservancy	[ ]	[ ]	[ ]	[ ]
Other animal welfare or conservation organization	[ ]	[ ]	[ ]	[ ]

22b. I have been a member of this organization for
(Please check [√] the box that best represents your response to this statement)

	Less than one year	1-2 years	3-5 years	6-10 years	More than 10 years
Organization that supports Trap-neuter and return	[ ]	[ ]	[ ]	[ ]	[ ]
The Audubon Society	[ ]	[ ]	[ ]	[ ]	[ ]
The Humane Society	[ ]	[ ]	[ ]	[ ]	[ ]
Alley Cat Allies	[ ]	[ ]	[ ]	[ ]	[ ]
American Bird Conservancy	[ ]	[ ]	[ ]	[ ]	[ ]
Other animal welfare or conservation organization ______________________________	[ ]	[ ]	[ ]	[ ]	[ ]

23. Do you currently feed outdoor only cats (not owned by you)?

- ☐ Yes
- ☐ No → *If you answered NO, please skip to question number **26**.*

24. If yes, how many cats do you typically feed?

- ☐ 1-5
- ☐ 6-10
- ☐ 11-20
- ☐ 21-30
- ☐ 31-50
- ☐ More than 50

25. Do you manage a cat colony?

- ☐ Yes
- ☐ No

26. How long have you lived at your current residence?

- ☐ Less than 1 year
- ☐ 1-5 years
- ☐ 6-10 years
- ☐ 11-15 years
- ☐ More than 15 years

27. In which year were you born? ☐
Y Y Y Y

28. I am

- ☐ Male
- ☐ Female

Thank you so much for taking the time to complete this survey. Your time is greatly appreciated. Please direct any questions about this survey to Dara Wald (352) 392-8372

This survey was approved by the University of Florida Institutional Review Board Protocol # 2010-U-0730.

Thank you for your participation in this survey!

If you have any additional thoughts about any of the topics in the survey, please share them here.

Dara Wald
University of Florida
PO Box 110430
Gainesville, FL 32611-0430
Phone: (352) 392-8372

NR-P

P
